# Assessing the association between tea intake and risk of dental caries and periodontitis: a two-sample Mendelian randomization study

**DOI:** 10.1038/s41598-024-54860-w

**Published:** 2024-02-27

**Authors:** Bilun Jin, Heng Chen, Peiqi Liu, Yijie Wang, Yi Guo, Chenxu Wang, Yue Jia, Rui Zou, Lin Niu

**Affiliations:** 1 Key Laboratory of Shaanxi Province for Craniofacial Precision Medicine Research, College of Stomatology, Xi’an Jiaotong University, Xi’an, China; 2 Clinical Research Center of Shaanxi Province for Dental and Maxillofacial Diseases, College of Stomatology, Xi’an Jiaotong University, Xi’an, China; 3 College of Stomatology, Xi’an Jiaotong University, Xi’an, China; 4 Department of Cardiology, The First Affiliated Hospital, Zhejiang University School of Medicine, Hangzhou, China

**Keywords:** Periodontitis, Dental public health

## Abstract

Tea is an indispensable beverage in people’s daily life. However, the relationship between tea intake and dental caries and periodontitis is controversial. We extracted datasets for tea intake and oral diseases from genome-wide association studies (GWASs) conducted by the UK Biobank and the Gene Lifestyle Interactions in Dental Endpoints consortium. We selected 38 single-nucleotide polymorphisms (SNPs) significantly associated with tea intake as instrumental variables (IVs) (*P* < 5.0 × 10^–8^). Mendelian randomization (MR) was performed to investigate the potential causality between tea intake and caries and periodontitis. Multivariable Mendelian randomization (MVMR) analyses were utilized to estimate causal effects of tea intake on risk of caries and periodontitis after adjusting for smoking, body mass index (BMI), and socioeconomic factors. The results showed that higher tea intake was suggestively associated with fewer natural teeth (β = − 0.203; 95% CI = 0.680 to 0.980; *P* = 0.029) and higher risk of periodontitis (OR = 1.622; 95% CI = 1.194 to 2.205; *P* = 0.002). After Bonferroni correction, the causality of tea intake on periodontitis remained significant. The significance of periodontitis disappeared after adjusting for the socioeconomic factors in MVMR (OR = 1.603; 95% CI = 0.964 to 2.666; *P* = 0.069). Tea intake had no association with risk of caries. Statistical insignificance of the heterogeneity test and pleiotropy test supported the validity of the MR study. Our results provide insight into the potential relationship between tea intake and oral diseases from a dietary lifestyle perspective, which may help prevent oral diseases.

## Introduction

Oral conditions affect 3.9 billion population worldwide, with dental caries, periodontitis, and tooth loss being the most common issues^1^. Dental caries is defined as chronic destruction of dental hard tissues produced by bacterial fermentation. Carious teeth are often characterized by demineralization and cavities^2^. Periodontitis is a chronic inflammatory disease caused by bacteria and spirochetes in the periodontal tissues^3^. The diagnosis of periodontitis is determined by the presence and severity of periodontal pockets, loss of clinical attachment, alveolar bone loss, or a combination of these factors^3–5^. It is one major reason for oral malodor and teeth loosening^6,7^. In the advanced stage of caries and periodontitis, tooth loss frequently occurs^8,9^. These outcomes affect not only esthetics, but also the functions to mastication and social development^10^. More importantly, these issues are closely connected with some systemic diseases^11^. For example, periodontitis is an independent risk factor for coronary heart disease^12,13^. Periodontitis is also associated with an increasing cancer mortality^14^. Diabetes, rheumatoid arthritis and some other chronic diseases are also closely related to periodontitis^15^. As for the aetiology of the oral diseases, various aspects are involved. The risk factors include unhealthy lifestyles, psychological distress, oral microbiome and even genetics^16,17^. Among them, dietary habits are intimately associated with oral diseases^2,18^. However, the association between beverages and oral diseases remains to be explored.

As one of the most consumed beverages globally, the potential effect of tea on oral diseases is still debatable. On the one hand, tea is beneficial to prevent oral diseases. As several studies manifested, dysregulation of oral microbiome would cause dental caries and periodontitis^19,20^. Epigallocatechin-3-gallate (EGCG), as a functional ingredient of tea, can widely inhibit the pathogenic bacteria of dental caries (*Streptococcus mutans, Actinomyces oris*) and periodontitis (*Porphyromonas gingivalis, Actinobacillus actinomycetemcomitans, Actinomyces israelii, Fusobacterium nucleatum, *etc*.*)^21^. Tea increases fluoride consumption which helps prevent dental caries^22^. In addition, a randomized clinical trial revealed that patients with periodontitis after scaling and root planning had lower probing depth and bleeding index after given 6-week green tea intake^23^. On the other hand, tea was regarded as a risk factor for caries because of the erosive effect on enamel^24^. A cross-sectional survey showed that one or more cups tea intake might be harmful to periodontitis^25^. High doses of caffeine in tea could accelerate bone loss and exacerbate the progression of periodontitis^26,27^. Until now, there remains controversy as to the causality of tea intake on oral diseases. Recognition of the causal link may advance the prevention and treatment for oral diseases.

Mendelian randomization (MR) is an application of genetic variation to infer whether phenotypic traits or exposures affect diseases or health-related outcomes. It is generally independent of confounders or the processes of disease^28^. Two-sample MR can explore causality between independent databases and save cost at the same time^29^. Therefore, two-sample MR enables more thorough understandings of the causal effect between exposure and outcome. Genetics plays important roles in tea intake and the causes of oral diseases. It has been demonstrated that genetic variables of tea intake are associated with a higher predicted perceived intensity of propylthiouracil and quinine^30^. People prefer bitterness and floral scents are more likely to drink tea^31^. In addition, variation in genetics could influence the status of oral health^32^. Therefore, MR analysis allows more comprehensive insights into the causality of tea intake on oral diseases.

Here, we performed a two-sample MR study to explore the relationship between tea intake and risk of dental caries and periodontitis based on relevant genome-wide association studies (GWASs). Further understanding of potential relationships between tea intake and dental caries and periodontitis is expected to provide new insights into the prevention of dental caries and periodontitis.

## Materials and methods

### Study design

**Figure 1:**
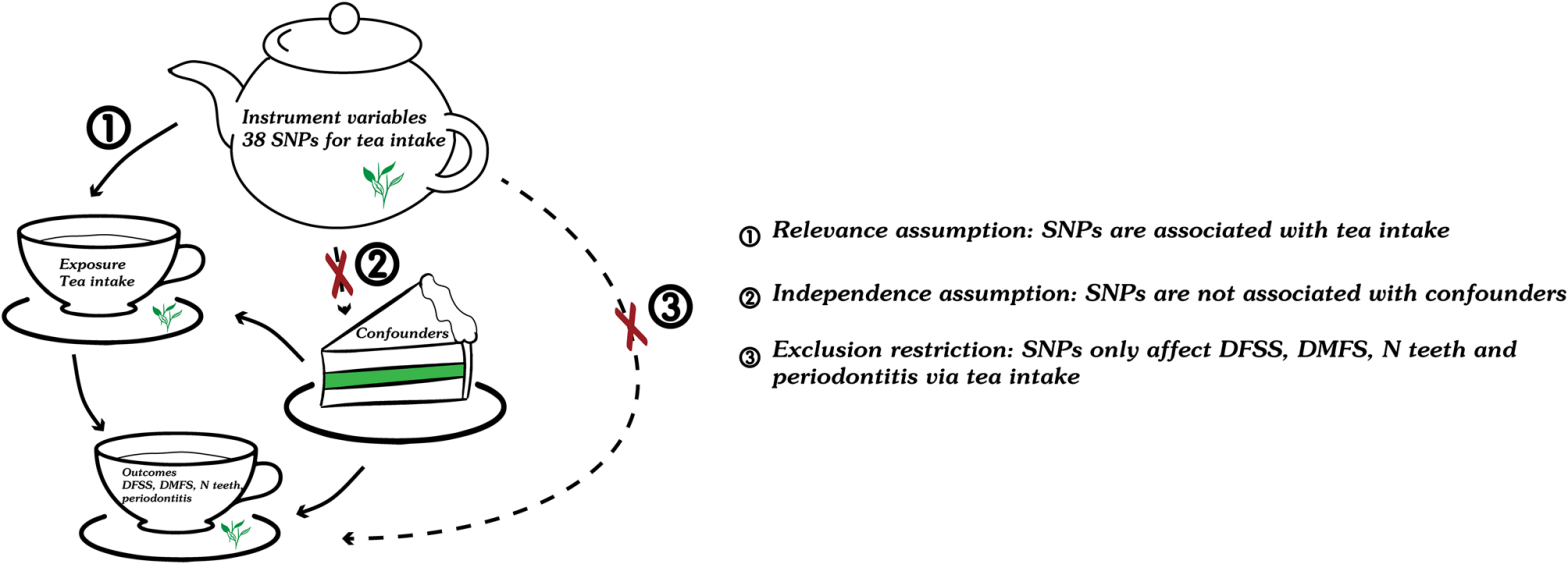
The illustrative diagram of this two-sample Mendelian randomization analysis. *Note:* DFSS, Decayed, Missing and Filled tooth Surfaces per available tooth surface; DMFS, Decayed, Missing and Filled tooth Surfaces; N teeth, Natural teeth remaining in the mouth.

The single-nucleotide polymorphisms (SNPs) as tea intake instrumental variables (IVs) and datasets for oral diseases were selected from two different GWASs^33,34^. The present two-sample MR study design was based on three essential assumptions (Fig. 1). Also, the Strengthening the Reporting of Observational Studies in Epidemiology Using Mendelian Randomization statement was followed by this study (Table S1). Additionally, smoking^35^ and body mass index (BMI)^36^ are detrimental factors for the risk of dental caries and periodontitis. We performed multivariable Mendelian randomization (MVMR) analyses to further estimate the casual effect of tea intake on the risk of caries and periodontitis after adjusting for smoking, BMI and socioeconomic factors including years of schooling and average total household income before tax.

The present study was based on the publicly available GWAS data and ethics consent had been obtained in the original studies^34,37^.

### GWAS data of tea intake

UK Biobank (UKB) is a large prospective study aims to investigate the role of genetics, environment and lifestyle in the causes of leading diseases. Data comes from 500,000 volunteers aged 40–69 in the United Kingdom^37^. We selected SNPs associated with tea intake from UKB which included 447,485 European participants (ID: ukb-b-6066). A touchscreen questionnaire was used to define how many cups of tea (black tea and green tea) the participants drank per day, and units of measurement were cups/day. As for quality control, participants were asked to confirm again if the answer > 20. And participants whose answer was less than 0 or more than 99 cups of tea each day were excluded^38^.

### GWAS data of caries and periodontitis

The datasets of caries and periodontitis were obtained from the published research, which was conducted by the Gene Lifestyle Interactions in Dental Endpoints (GLIDE) consortium^34^. In the studies of GLIDE, only European ancestry individuals were retained. The traits covered DMFS (Decayed, Missing and Filled tooth Surfaces) and DFSS (Decayed, Missing and Filled tooth Surfaces per available tooth surface) as the experience of caries, as well as N teeth (number of natural teeth) and the presence of periodontitis. Participants with no teeth were excluded from the analysis in GLIDE. See more details of information of exposure and outcome datasets in Table S2. In order to perform MVMR analyses, summary data on smoking and BMI were obtained from the GWASs in UKB (ID: ukb-b-223) and the Genetic Investigation of Anthropometric Traits consortium (ID: ieu-a-835), data on socioeconomic factors contained years of schooling (ID: ieu-a-1239) and average total household income before tax (ID: ukb-b-7408).

### Instrumental variable selection

Follow the steps below, IVs were extracted from UKB. SNPs associated with routine tea intake were identified at the genome-wide significance level (*P* < 5 × 10^−8^). To select independent SNPs as IVs, a strict clumping procedure was performed, setting the linkage disequilibrium (LD) coefficient r^2^ to be less than 0.001 in a 10,000-kb window referring to the European 1000G panel^39^. Then we used the PhenoScanner tool to check whether any of the selected SNPs were associated with potential confounders, such as intake of sugar, sweeteners or milk, smoking, diabetes, BMI and socioeconomic factors. SNPs with a minor allele frequency less than 0.01 should be excluded to avoid the statistical bias from the original GWAS due to the low confidence. Finally, the R^2^ and F statistic were also calculated. F greater than 10 means that the study would be influenced by limited bias from weak instruments^40^.

### Statistical analysis

The inverse-variance-weighted (IVW) analysis was considered as a fundamental approach with pooled Wald ratios^41^. Other approaches including weighted median, MR Egger, simple median, MR PRESSO and MR RAPS were also exploited to compensate for the loss of testing horizontal pleiotropic effects from IVs to oral diseases. (1) Weighted median: The weighted median analysis can provide consistent causal estimates, providing that more than half of weight derives from valid SNPs^42^; (2) MR Egger: The method allows free evaluation of the intercept (not set to zero) as an indicator of average pleiotropic bias and gives a consistent estimate when all genetic variants are invalid IVs^43^; (3) Simple median: This is also a valid approach to analyze the sensitivity of IVs^42^; (4) MR PRESSO: It can identify possible outliers and provide causal estimates after removal of outlying IVs^44^; (5) MR RAPS: MR RAPS can overcome the bias of weak IVs and the effects of systematic and idiosyncratic pleiotropy for robustness^45^. When dealing with multiple outcomes, the Bonferroni method was utilized to adjust the significance thresholds. *P*-value that lower than the adjusted threshold indicated statistical significance. Furthermore, *P*-value less than 0.05 but no less than the Bonferroni-adjusted significance level would be considered to be suggestively significant.

In order to complete the sensitivity analysis, the Cochran’s Q value and I^2^ were used to assess the heterogeneity between various SNP estimates^46^. Scatter plots, funnel plots and leave-one-out analysis were also executed to estimate causality and heterogeneity^47,48^. *P*-value of MR Egger intercept is a judgement index of pleiotropy. The indicate pleiotropy was set as *P*-value < 0.05. Next, MR PRESSO was applied to find outliers and estimate the casual effects without outliers, if any.

Finally, we performed a multivariable two-sample MR analysis between tea intake and dental caries and periodontitis after adjustment for smoking, BMI and socioeconomic factors (years of schooling and average total household income before tax). The statistical power was calculated via an online calculator named mRnd (https://shiny.cnsgenomics.com/mRnd/). All data were analyzed by R (Version 3.1.5), combined with R packages “TwoSampleMR”, “mr.raps.”, “MR-PRESSO”.

### Ethical approval and consent to participate

Ethical approval had been obtained in all original studies. The OpenGWAS Database is a publicly available dataset, and GWAS of oral diseases complied with all relevant ethical regulations, including the Declaration of Helsinki, and ethical approval for data collection and analysis was obtained by each study from local boards. All participants provided their written informed consent in the original studies.

## Results

We identified 41 SNPs associated with routine tea intake at the genome-wide significance level (*P* < 5 × 10^−8^). Because of the possible link to smoking and type II diabetes, we excluded rs1453548 and rs9937354 among the selected SNPs. In addition, rs149805207 with a minor allele frequency of < 0.01 was also excluded to avoid potential statistical bias from the original GWAS since the low confidence. In total, 38 SNPs were recognized as eligible IVs for the present two-sample MR study (Table S3). The F statistic for each IV was above 10, and R^2^ value summed up to 3.5%.

**Figure 2:**
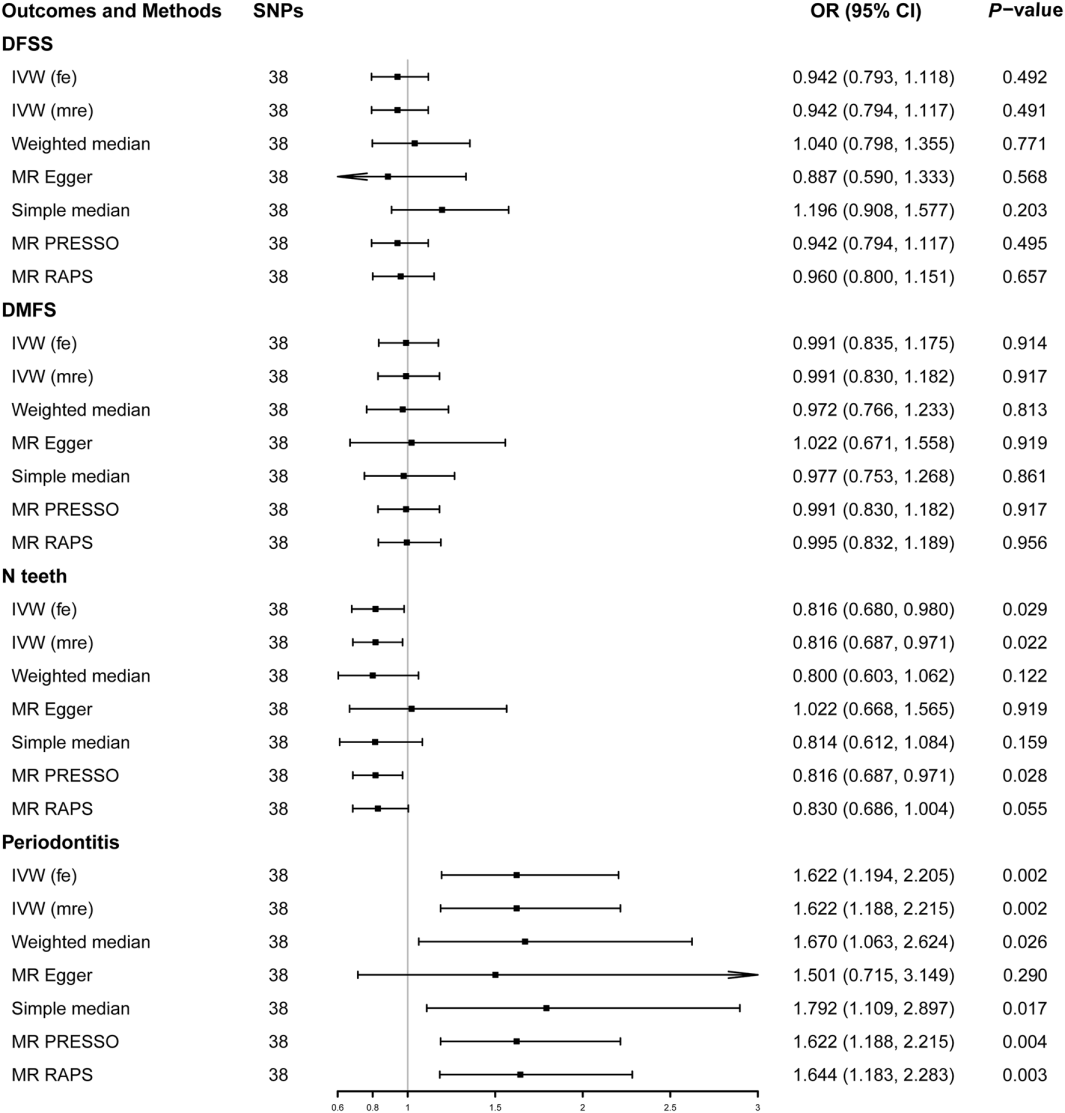
Estimates of tea intake on the risk of caries and periodontitis. *Note:* DFSS, Decayed, Missing and Filled tooth Surfaces per available tooth surface; DMFS, Decayed, Missing and Filled tooth Surfaces; N teeth, Natural teeth remaining in the mouth; OR, Odds ratio; CI, Confidence interval; IVW, inverse-variance weighted; IVW (fe), fixed effects inverse-variance weighted; IVW (mre), multiplicative random effect inverse-variance weighted; MR-PRESSO, MR-pleiotropy residual sum and outlier; MR-RAPS, MR-robust adjusted profile score.

**Figure 3:**
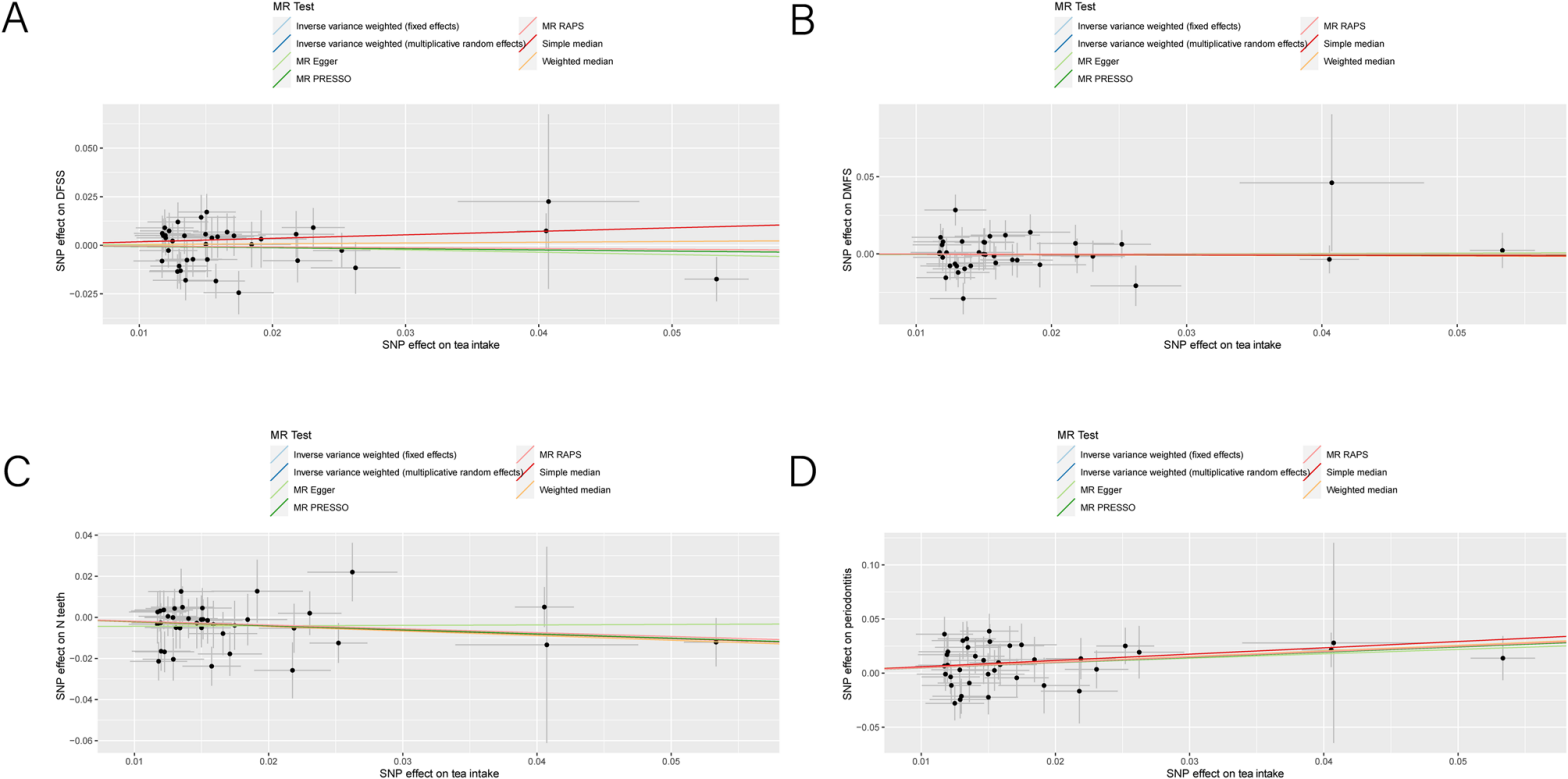
Scatter plot of the MR estimates for the association of tea intake with risk of caries (**A**, **B**) and periodontitis (**C**, **D**). Each point in the scatter plot represents an SNP. The effect of one SNP on tea intake is showed on the horizontal axis, and the effect of the same SNP on oral diseases is on the vertical axis. The vertical and horizontal lines around each point show the 95% confidence interval (CI) for each SNP. The slope of the colorful solid line in the plot is the Mendelian randomization (MR) estimates via different methods. *Note:* DFSS, Decayed, Missing and Filled tooth Surfaces per available tooth surface; DMFS, Decayed, Missing and Filled tooth Surfaces; N teeth, Natural teeth remaining in the mouth; MR-PRESSO, MR-pleiotropy residual sum and outlier; MR-RAPS, MR-robust adjusted profile score.

At a significance threshold of 0.05, our results showed that tea intake frequency was negatively associated with N teeth (β = − 0.203; 95% CI = 0.680 to 0.980; *P* = 0.029) and positively associated with the presence of periodontitis (odds ratio (OR) = 1.622; 95% CI = 1.194 to 2.205; *P* = 0.002). Moreover, tea intake had no association with DFSS or DMFS based on the fixed-effect IVW model. MR PRESSO found no outliers in each outcome dataset. The associations remained consistent in supplementary methods based on most statistical models, indicating the robustness of the results. The associations of tea intake with N teeth remained directionally consistent across weighted median, simple median and MR RAPS methods, albeit with wider CIs. Both the associations with N teeth and periodontitis were lower with wider 95% CIs in MR Egger method (Figs. 2 and 3). Following Bonferroni correction (*P* = 0.05/4 = 0.0125 [for the 4 exposure-outcome pairs]), the association between tea intake and periodontitis remained statistically significant. However, the association between tea intake and N teeth was not significant at a Bonferroni-adjusted threshold.

As for sensitivity analysis, there was no sufficient evidence of heterogeneity among all the analyses as the *P*-values for Cochran’s Q and I^2^ manifested (Table 1). The *P*-value for MR Egger intercept was higher than 0.05 in each group, which demonstrated no significant horizontal pleiotropy in this study (Table 1). No substantial difference appeared in the estimated causal effect when repeating the analysis without individual SNP (Figs. S1–S4). Also, the funnel plots were roughly symmetric (Figs. S5–S8). Multivariable MR indicated that tea intake was a potential harmful factor for natural teeth in mouth (β = − 0.220; 95% CI = 0.664 to 0.970; *P* = 0.023) and periodontitis (OR = 1.453; 95% CI = 1.060 to 1.991;* P* = 0.020), independently of BMI and smoking (Table 2). The association between tea intake and natural teeth in mouth (β = − 0.309; 95% CI = 0.541 to 0.996; *P* = 0.047) persisted after adjusting for years of schooling and average total household income before tax. The causal association of tea intake on periodontitis (OR = 1.603; 95% CI = 0.964 to 2.666; *P* = 0.069) was, however, attenuated after adjusting for the two socioeconomic factors, indicating that causality might be partly mediated by socioeconomic factors (Table 3).

**Table 1:** Results of potential pleiotropy and heterogeneity assessments after excluded pleiotropic SNP on associations between tea intake and risk of caries and periodontitis.

Outcomes	Heterogeneity		Pleiotropy	
	*P*-value for Cochran’s Q	Cochran’s Q statistic	I^2^ (%)	*P*-value for MR-Egger intercept
DFSS	0.479	36.784	0	0.753
DMFS	0.366	39.328	6	0.873
N teeth	0.636	33.461	0	0.259
Periodontitis	0.417	38.159	3	0.822

DFSS, Decayed, Missing and Filled tooth Surfaces per available tooth surface; DMFS, Decayed, Missing and Filled tooth Surfaces; N teeth, Natural teeth remaining in the mouth.

**Table 2:** Estimates of tea intake on the risk of caries and periodontitis after adjusting for smoking and BMI.

Outcomes	Beta	SE	OR (95% CI)	*P*-value
DFSS	− 0.034	0.097	0.967 (0.799, 1.171)	0.730
DMFS	0.016	0.089	1.013 (0.839, 1.224)	0.890
N teeth	− 0.220	0.097	0.802 (0.664, 0.970)	0.023
Periodontitis	0.373	0.161	1.453 (1.060, 1.991)	0.020

DFSS, Decayed, Missing and Filled tooth Surfaces per available tooth surface; DMFS, Decayed, Missing and Filled tooth Surfaces; N teeth, Natural teeth remaining in the mouth; Beta, estimate of the effect of the association; SE, standard error; OR, Odds ratio; CI, Confidence interval.

**Table 3:** Estimates of tea intake on the risk of caries and periodontitis after adjusting for years of schooling and average total household income before tax.

Outcomes	Beta	SE	OR (95% CI)	*P*-value
DFSS	− 0.296	0.159	0.744 (0.545, 1.016)	0.063
DMFS	− 0.106	0.163	0.899 (0.653, 1.238)	0.515
N teeth	− 0.309	0.155	0.734 (0.541, 0.996)	0.047
Periodontitis	0.472	0.260	1.603 (0.964, 2.666)	0.069

DFSS, Decayed, Missing and Filled tooth Surfaces per available tooth surface; DMFS, Decayed, Missing and Filled tooth Surfaces; N teeth, Natural teeth remaining in the mouth; Beta, estimate of the effect of the association; SE, standard error; OR, Odds ratio; CI, Confidence interval.

The study had strong power (100%, over 80%) to detect the causal effect of tea consumption on OR for periodontitis. The power of β for DFSS, DMFS and N teeth cannot be calculated due to the lack of β_OLS_.

## Discussion

Tea is popularly consumed worldwide, next to water^49^. Dental caries and periodontitis are chronic diseases without self-limit, they have been recognized as a common public health challenge^50^. However, the relationship between tea intake and dental caries and periodontitis continues to be controversial. We performed a two-sample MR study to explore the causal associations of tea intake with dental caries and periodontitis based on various GWASs. We found that incremental tea intake may reduce remaining natural teeth in mouth and augment the risk of periodontitis.

The most novel finding in our study is the positive correlation between tea intake and presence of periodontitis. Some potential mechanisms are presumed. So far, there is no direct evidence that tea causes periodontitis. However, some cross-sectional studies have shown a strong association between caffeine and periodontitis^51,52^. As we known, caffeine is a rich constituent in tea^53^. An animal experiment found that caffeine can accelerated alveolar bone loss in modeled periodontitis rats^26^. In addition, the hot tea may deteriorate the pathogenicity of smoking toward periodontitis^25^. Tea consumption might also indirectly cause periodontitis by inducing systemic diseases. For instance, green tea drinking was associated with an increased risk of type II diabetes in Chinese adults^54^. In general, further research regarding the causal relationship between tea intake and periodontitis is still needed.

Our study also illustrated that tea intake appeared to be not conducive to retain natural teeth in mouth. This could be a complement to the forementioned results, because untreated or abortively treated periodontitis always leads to tooth loss^55,56^. Nonetheless, opposite result was found in a cross-section survey in the effect of tea on tooth loss. The survey from Japan showed that drink more than one cup green tea per day could decrease the possibility of tooth loss^57^. The difference may come from types of tea^58^. Green tea contains more catechins (such as EGCG) playing antibiotic functions, comparing to black tea^59^. During the fermentation process of black tea, catechins level decreased gradually and caffeine concentrated double dry weight after a 85% fermentation^53^. The exposure population used in this study was Europeans, who prefer black tea, whereas Asians prefer green tea in comparison. This might be one of the reasons for the differences among the studies, which did not affect the validity of our study.

Our result showed that there was no significant evidence to support a relationship between tea intake and caries, which was consistent with a previous meta-analysis^60^. In spite of some green tea extract, especially EGCG was indicated benefit to caries prevention^61^, and fluoride in tea could protect teeth theoretically^62^, tea has erosive effect on enamel definitely. Even with buffering effects of saliva, the black tea pH of 4.8 might result in the demineralization of enamel (the threshold is the pH of 5.5)^24^. In this MR analysis, the effect of tea intake on dental caries was invalid, possibly due to the dual effect of tea consumption.

Some strengths should be identified in our study. Using MR analysis can reduce some confounders and avoid reverse causality compared with traditional cross-section studies. The population of exposure and outcome datasets are both European, which can reduce the ethnic differences affecting the polymorphism of genes. Moreover, our study assessed the heterogeneity via Cochran’s Q, with scatter plots, funnel plots and leave-one-out analysis as complement. MR Egger and MR PRESSO were utilized to test the horizontal pleiotropy. Multivariable MR also help to estimate the causal effect of tea intake on risk of caries and periodontitis, after adjusting for smoking, BMI and socioeconomic factors. Whereas, some limitations exist in this study. Firstly, the research objects used in our study were all Europeans, which may limit the extrapolation of research results. Secondly, no clear coefficient of association between tea intake and caries or remaining teeth in mouth has been published in any observational study, so it is currently difficult to evaluate the power of first three estimates. Thirdly, due to the lack of data what resulted in tooth loss, we could not assess whether the association between genetically predicted tea intake and tooth loss differs among various reasons, such as caries, periodontitis and even trauma. Finally, more explicit mechanisms on how tea intake acts on caries and periodontitis should be studied in the future.

## Conclusions

Collectively, our study manifested that frequent tea consumption may increase risk of periodontitis through genetic evidence. We suggest tea intake as a novel target in the prevention of periodontitis and tooth loss. Meanwhile, we will further explore relevant mechanisms in the future.

### Supplementary Information

Supplementary Legends.

Supplementary Figure S1.

Supplementary Figure S2.

Supplementary Figure S3.

Supplementary Figure S4.

Supplementary Figure S5.

Supplementary Figure S6.

Supplementary Figure S7.

Supplementary Figure S8.

Supplementary Table S1.

Supplementary Table S2.

Supplementary Table S3.

## Data Availability

Data on tea intake (ID: ukb-b-6066), BMI (ID: ukb-b-223), smoking (ID: ieu-a-835), years of schooling (ID: ieu-a-1239) and average total household income before tax (ID: ukb-b-7408) are available in the IEU open GWAS project, (https://gwas.mrcieu.ac.uk/). Data on oral diseases including caries, tooth loss and periodontitis are included in this published article (Genome-wide analysis of dental caries and periodontitis combining clinical and self-reported data, doi: 10.1038/s41467-019-10630-1) and its supplementary information files (https://data.bris.ac.uk/data/dataset/2j2rqgzedxlq02oqbb4vmycnc2). The datasets used during the current study are available from the corresponding author on reasonable request.
