# Supplementary material for: Assessing the association between tea intake and risk of dental caries and periodontitis: a two-sample Mendelian randomization study
Source: Sci Rep. 2024 Feb 27;14:4728. doi: 10.1038/s41598-024-54860-w (PMC10899219; doi:10.1038/s41598-024-54860-w)
Supplement: Supplementary file 1 — Supplementary Legends. [file 41598_2024_54860_MOESM1_ESM.docx]

**Supplementary materials**

**Fig S1.** Leave-one-out analysis of individual and combined SNP MR estimates for tea intake on DFSS.

**Fig S2.** Leave-one-out analysis of individual and combined SNP MR estimates for tea intake on DMFS.

**Fig S3.** Leave-one-out analysis of individual and combined SNP MR estimates for tea intake on N teeth.

**Fig S4.** Leave-one-out analysis of individual and combined SNP MR estimates for tea intake on periodontitis.

**Fig S5.** Funnel plot to evaluate heterogeneity of MR estimates for the causality of tea intake on DFSS.

**Fig S6.** Funnel plot to evaluate heterogeneity of MR estimates for the causality of tea intake on DMFS.

**Fig S7.** Funnel plot to evaluate heterogeneity of MR estimates for the causality of tea intake on N teeth.

**Fig S8.** Funnel plot to evaluate heterogeneity of MR estimates for the causality of tea intake on periodontitis.

**Table S1.** STROBE-MR Checklist.

**Table S2.** Information of exposure and outcome datasets in detail.

**Table S3.** The characteristics of the selected SNPs on tea intake.
