# Supplementary figures and images for: Assessing the association between tea intake and risk of dental caries and periodontitis: a two-sample Mendelian randomization study
Source: Sci Rep. 2024 Feb 27;14:4728. doi: 10.1038/s41598-024-54860-w (PMC10899219; doi:10.1038/s41598-024-54860-w)

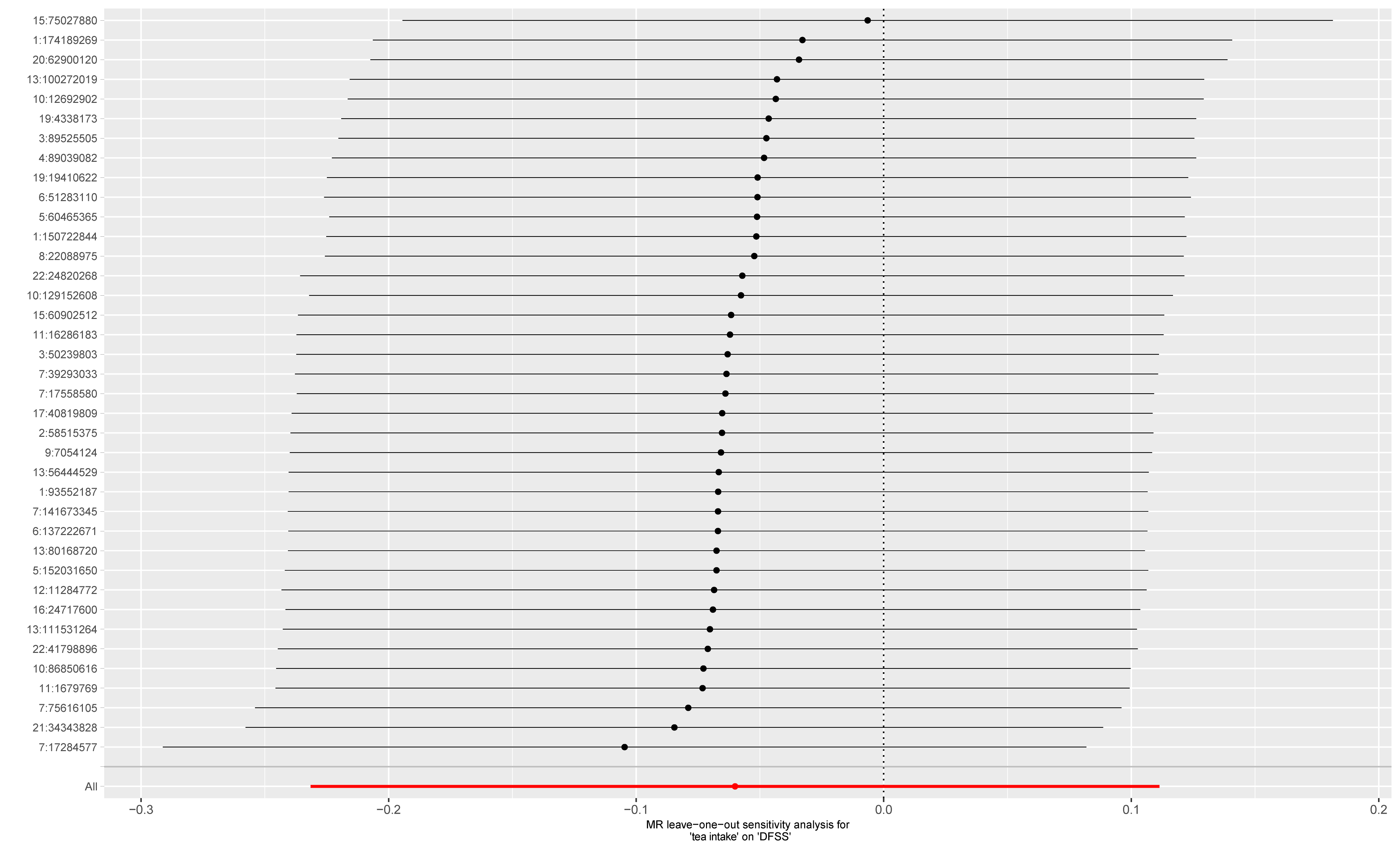

Supplement: Supplementary file 2 — Supplementary Figure S1. [file 41598_2024_54860_MOESM2_ESM.tif]

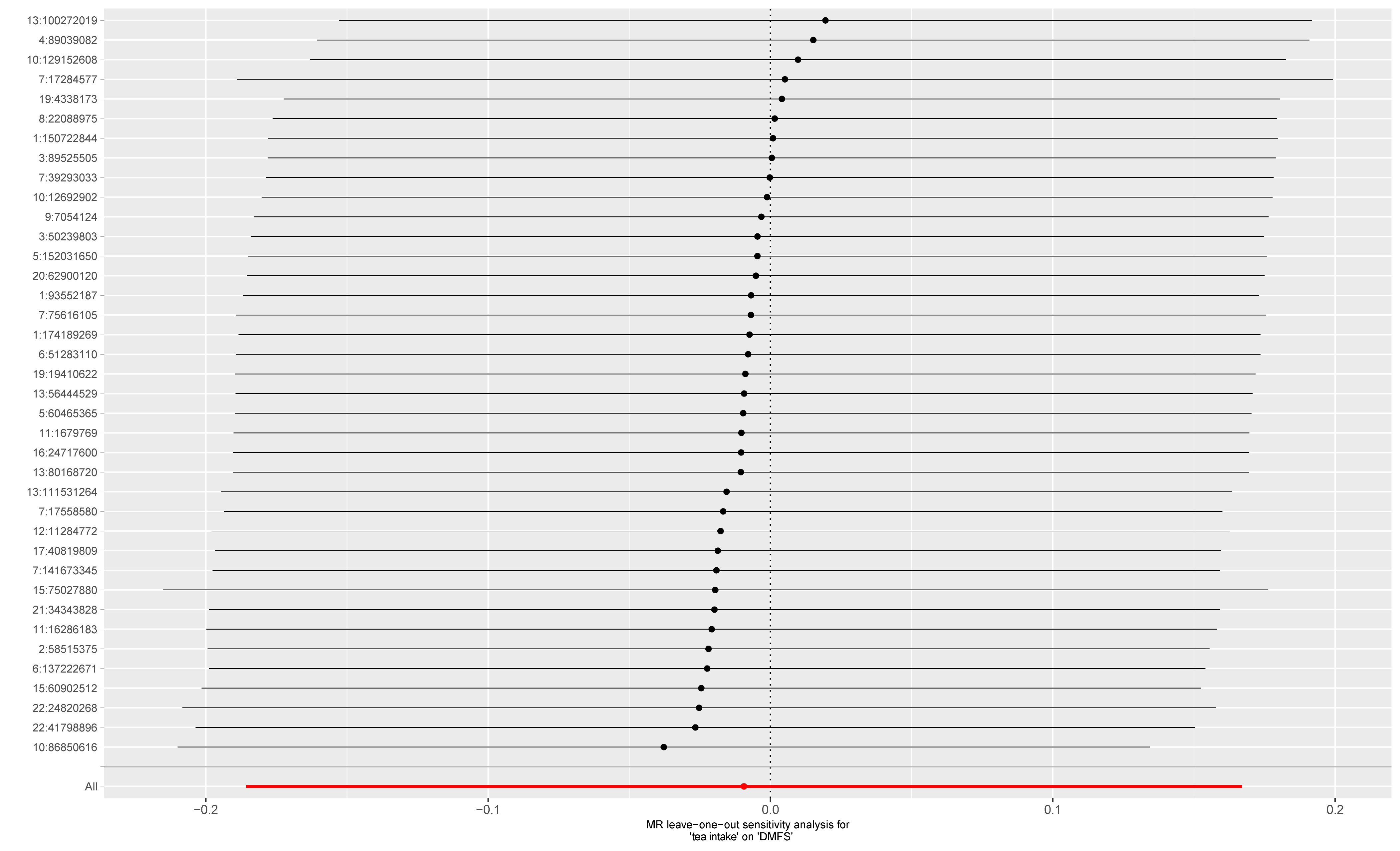

Supplement: Supplementary file 3 — Supplementary Figure S2. [file 41598_2024_54860_MOESM3_ESM.tif]

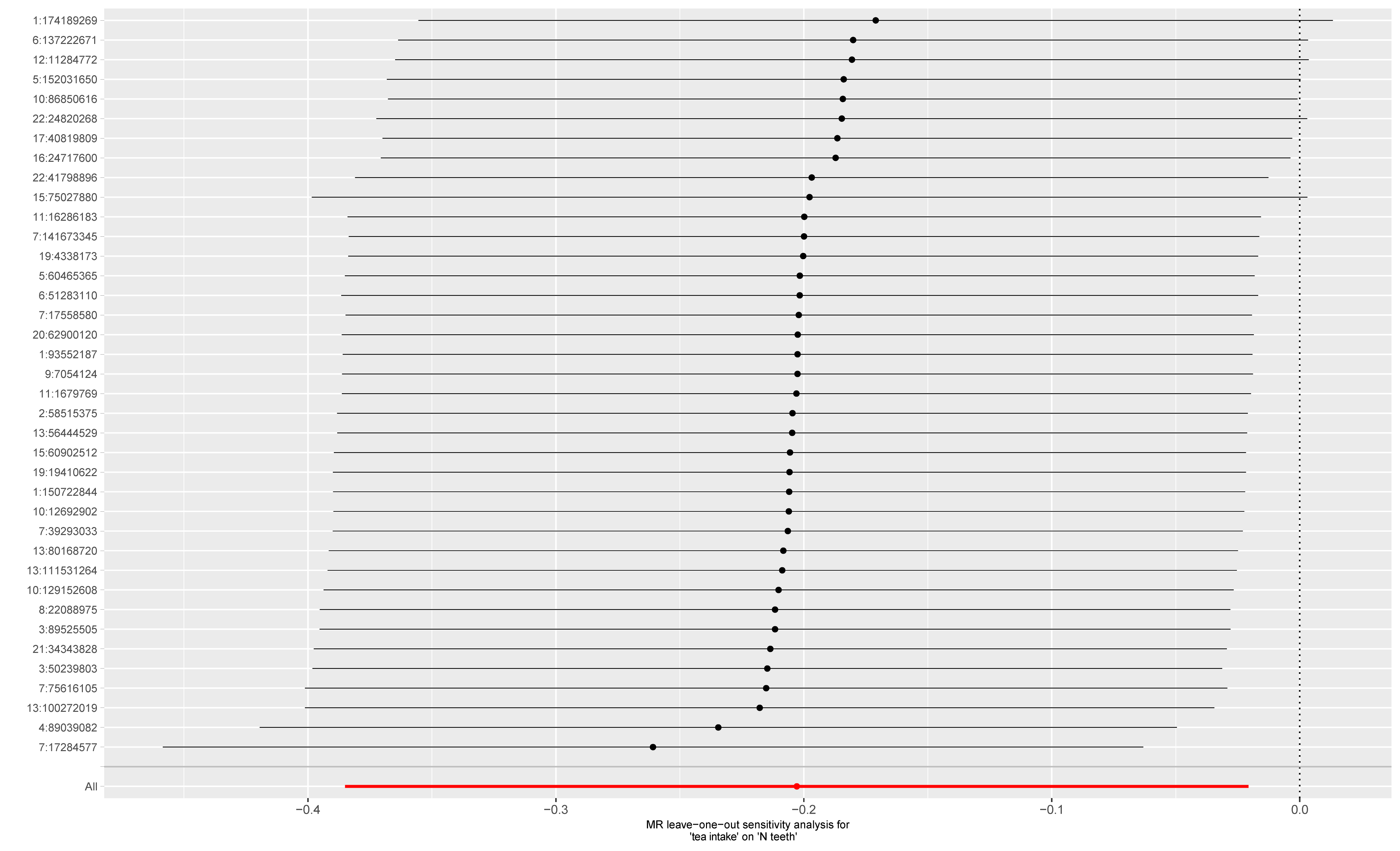

Supplement: Supplementary file 4 — Supplementary Figure S3. [file 41598_2024_54860_MOESM4_ESM.tif]

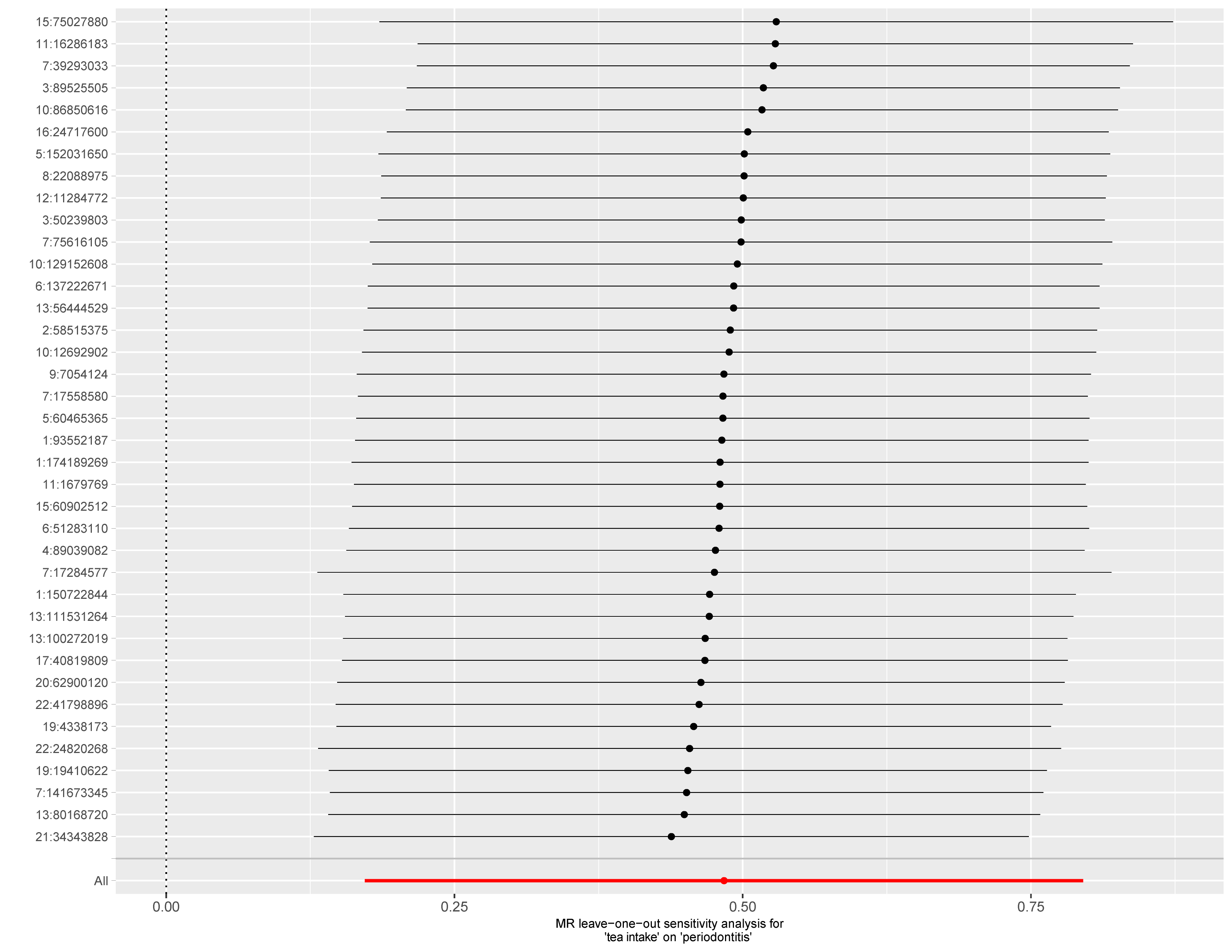

Supplement: Supplementary file 5 — Supplementary Figure S4. [file 41598_2024_54860_MOESM5_ESM.tif]

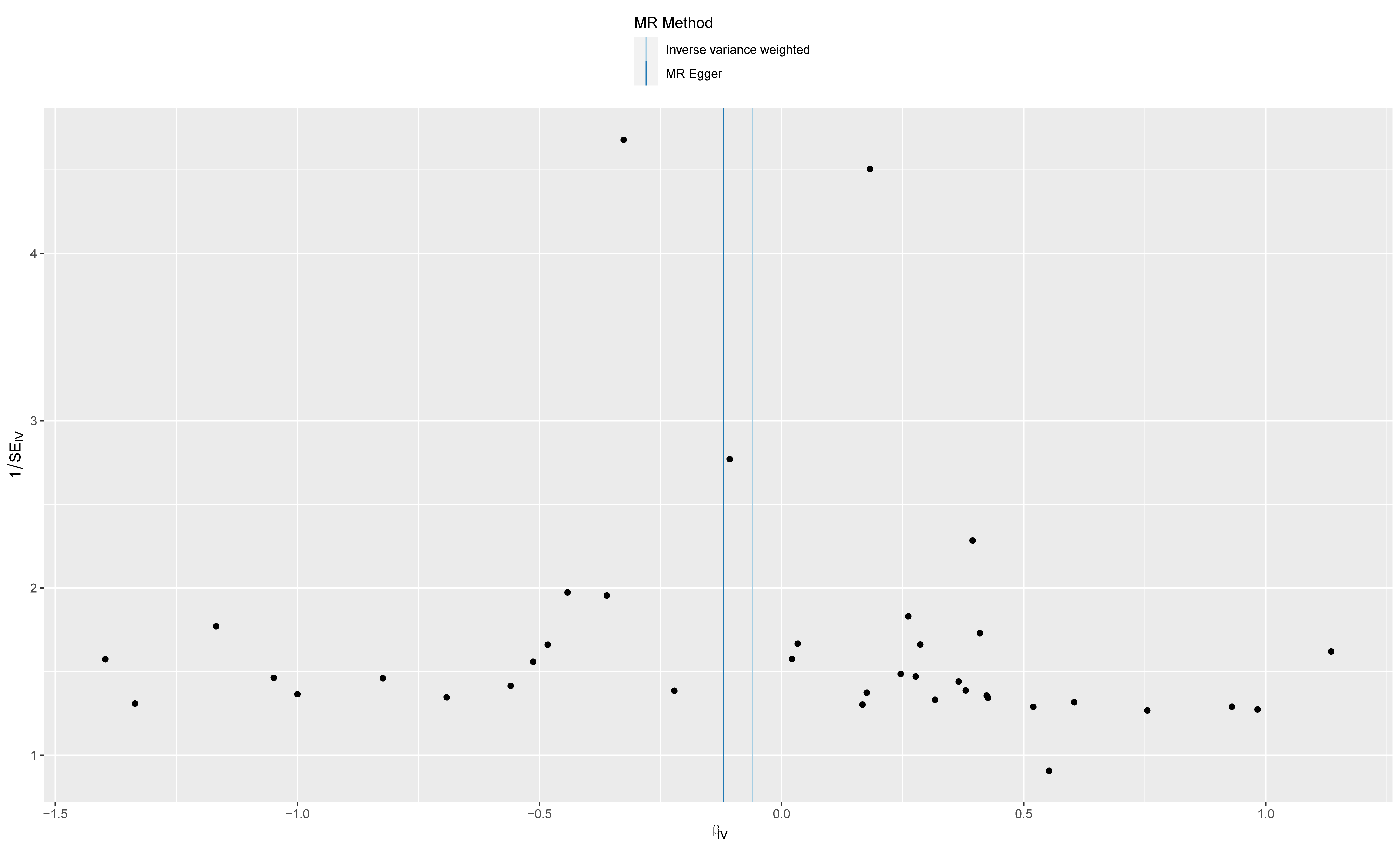

Supplement: Supplementary file 6 — Supplementary Figure S5. [file 41598_2024_54860_MOESM6_ESM.tif]

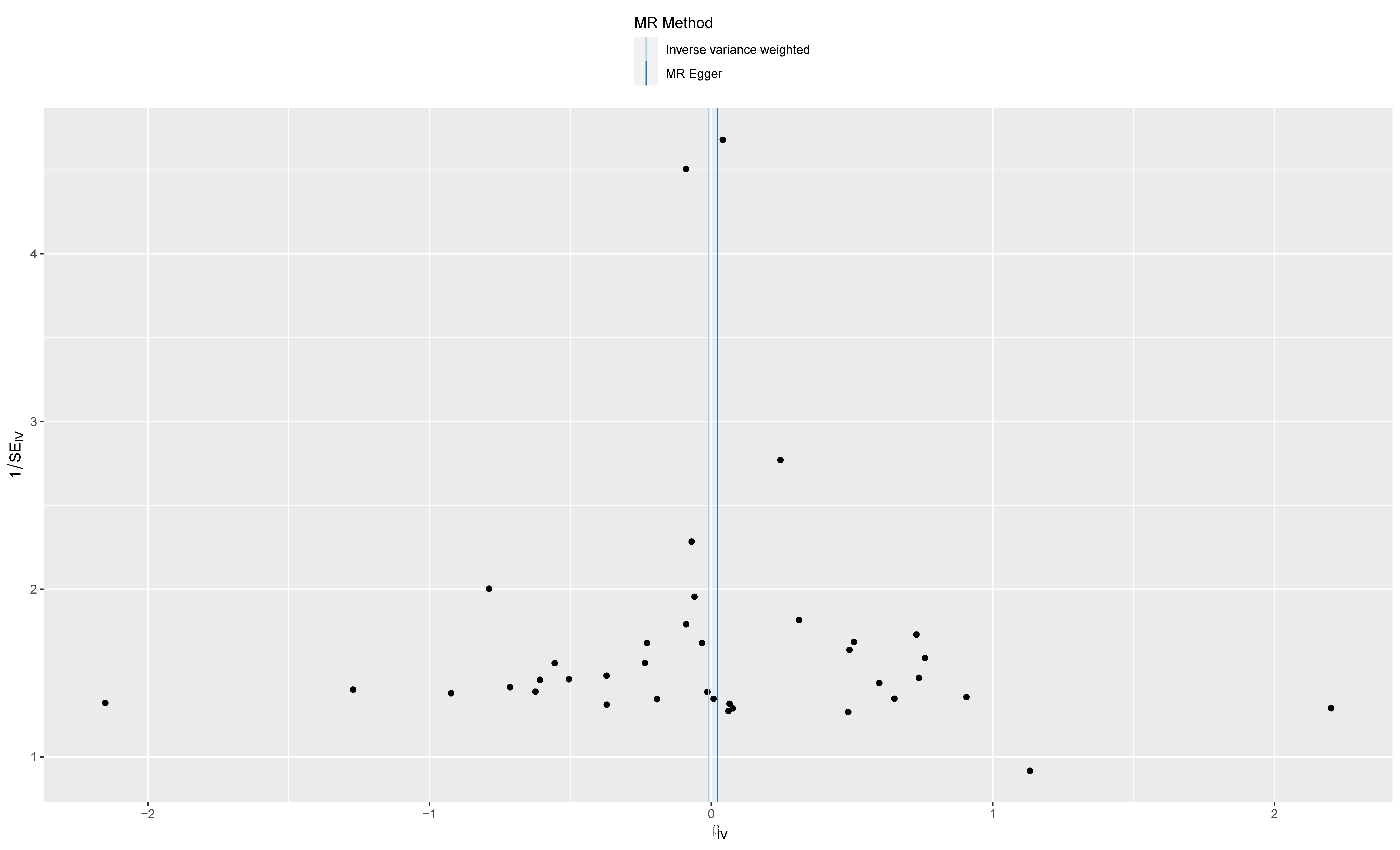

Supplement: Supplementary file 7 — Supplementary Figure S6. [file 41598_2024_54860_MOESM7_ESM.tif]

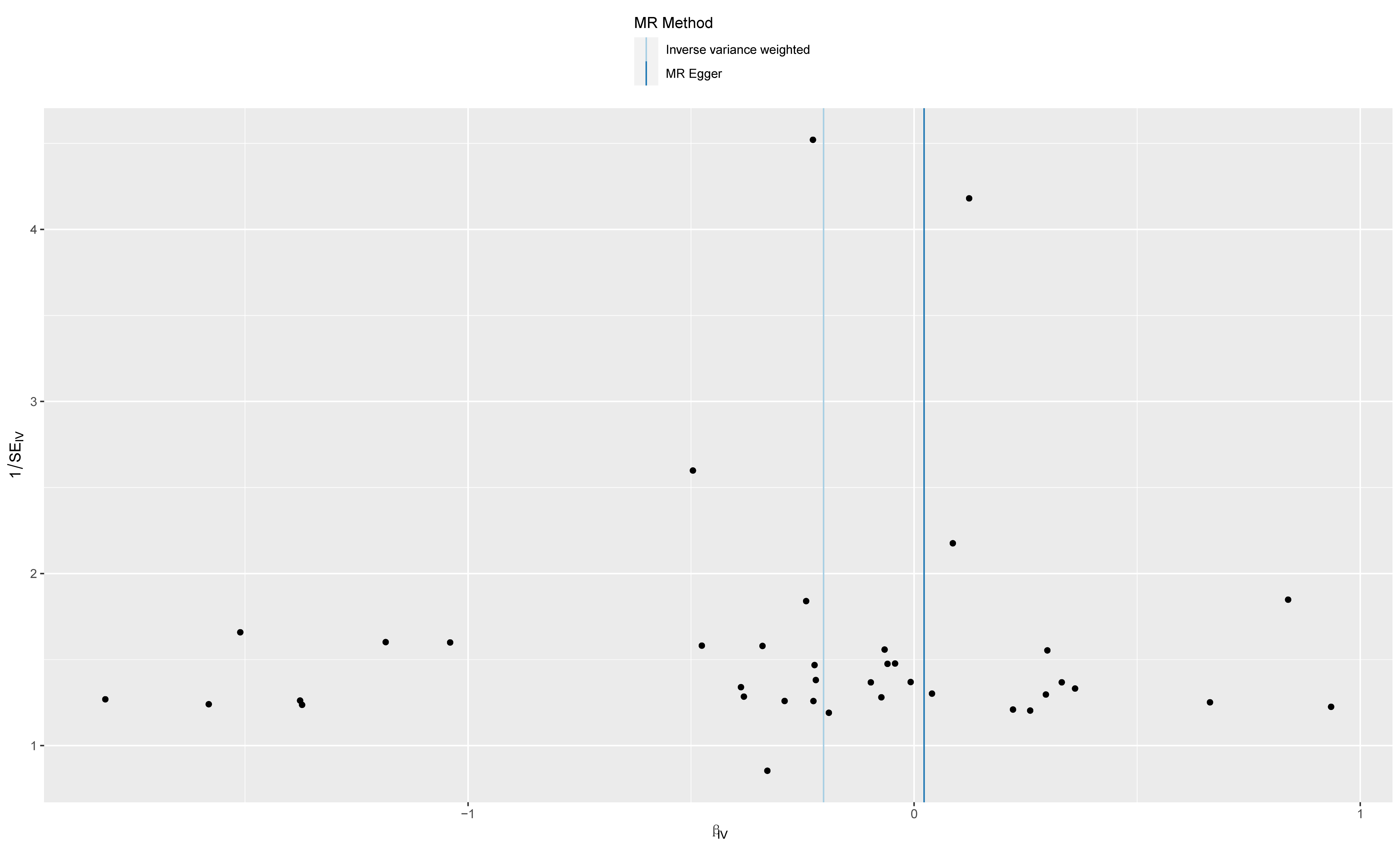

Supplement: Supplementary file 8 — Supplementary Figure S7. [file 41598_2024_54860_MOESM8_ESM.tif]

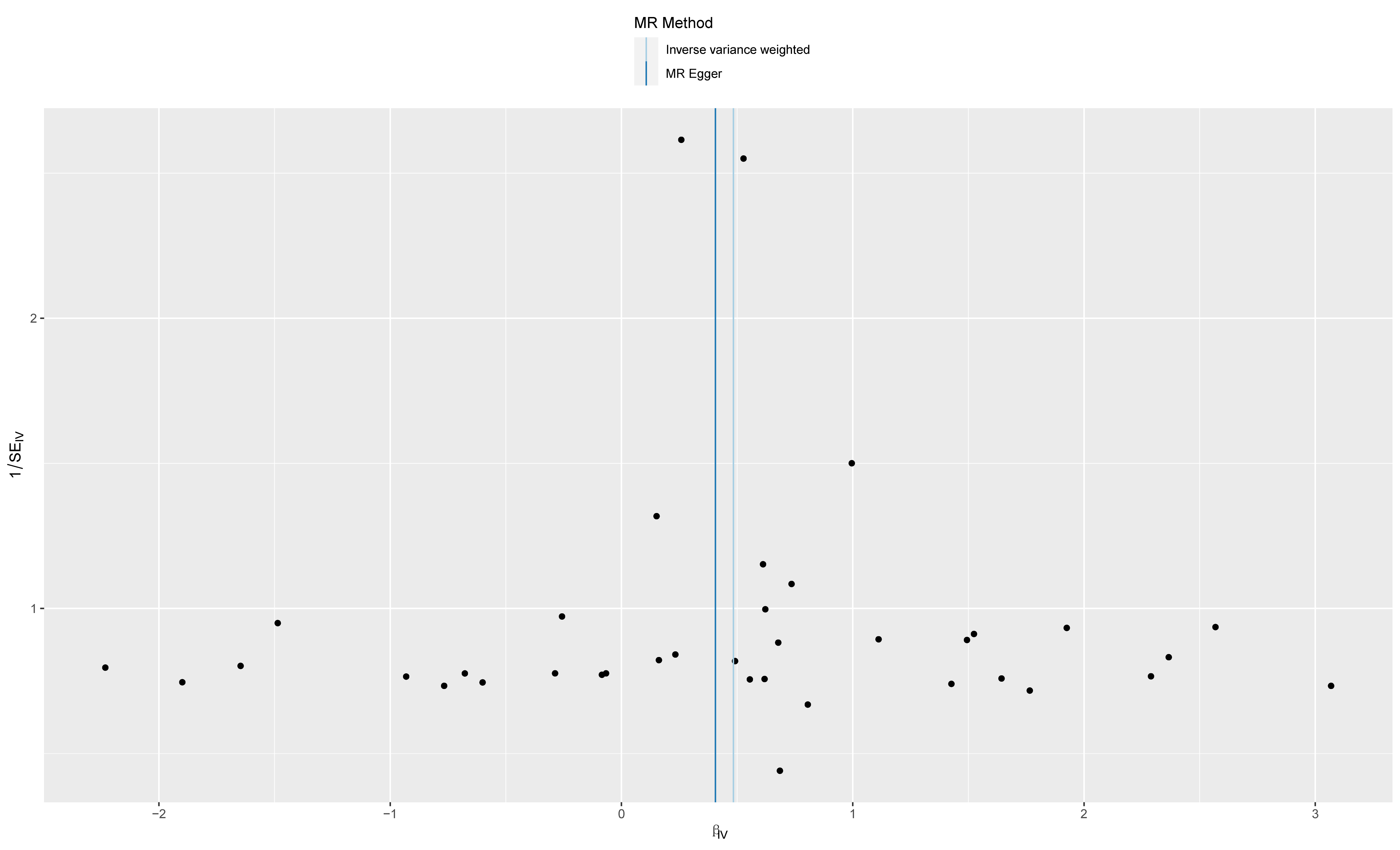

Supplement: Supplementary file 9 — Supplementary Figure S8. [file 41598_2024_54860_MOESM9_ESM.tif]
